# Transfer of brotizolam, periciazine, and sulpiride in cord blood and breast milk, and alprazolam in breast milk: a case report

**DOI:** 10.1186/s40780-022-00241-2

**Published:** 2022-04-01

**Authors:** Jumpei Saito, Yoshiyuki Tachibana, Yuka Sano Wada, Naho Yakuwa, Hiroyo Kawasaki, Tomo Suzuki, Haruhiko Sago, Akimasa Yamatani, Atsuko Murashima

**Affiliations:** 1 Department of Pharmacy, National Center for Child Health and Development, Tokyo, Japan; 2 Division of Infant and Toddler Mental Health, Department of Psychosocial Medicine, National Center for Child Health and Development, Tokyo, Japan; 3 Division of Neonatology, Center for Maternal-Fetal, Neonatal, and Reproductive Medicine, National Center for Child Health and Development, Tokyo, Japan; 4 Japan Drug Information Institute in Pregnancy, National Center for Child Health and Development, Tokyo, Japan; 5 Division of Maternal Medicine, Center for Maternal-Fetal, Neonatal and Reproductive Medicine, National Center for Child Health and Development, Tokyo, Japan; 6 Division of Obstetrics, Center for Maternal-Fetal, Neonatal and Reproductive Medicine, National Center for Child Health and Development, Tokyo, Japan

**Keywords:** Psychiatry, Psychotherapy, Pregnancy, Lactation

## Abstract

**Background:**

A high prevalence of mental disorders including depression, anxiety, somatoform, and dissociative disorder is reported during pregnancy, however, information on the transfer of antipsychotics across the placenta and into breast milk is limited. We evaluated brotizolam, periciazine and sulpiride in cord blood, maternal serum, and breast milk, and alprazolam in breast milk.

**Case presentation:**

A 38-year-old woman with dissociative disorder was treated with brotizolam, propericiazine, and sulpiride during pregnancy and lactation, and alprazolam during lactation. The drug concentration ratios for both cord blood and maternal serum were 33.3 and 61.5% for brotizolam and sulpiride, respectively, and periciazine (metabolite of propericiazine) was not detected in the cord blood. In breast milk, alprazolam (0.9 ng/mL), sulpiride (445.8 ng/mL), and periciazine (0.3 ng/mL) concentrations were noted at 7.5 h after the last dose on postpartum, whereas brotizolam was not detected. The relative infant doses via breast milk were 1.4, 2.7 and 0.02% of the maternal daily dose, respectively. The neonate had no congenital anomalies and did not experience any severe withdrawal symptoms after birth.

**Conclusion:**

Use of brotizolam, propericiazine, and sulpiride during pregnancy and lactation, and use of alprazolam during lactation were acceptable in this case.

## Background

A high prevalence of mental disorders including depression, anxiety, somatoform, and dissociative disorder is reported during pregnancy [1], and 10–25% of pregnant women with these disorders are prescribed antidepressants, such as benzodiazepines or benzodiazepine-like hypnotic drugs [2, 3], or antipsychotic drugs, such as phenothiazine-, butyrophenone-, and benzamide-derivative agents [4]. Benzodiazepines have been associated with increased risks of preterm birth, low Apgar score, and neonatal respiratory distress syndrome [5]. Although whether antipsychotic agents can affect pregnancy outcome is unclear [6], withdrawal symptoms in neonates exposed to antipsychotics during pregnancy have been reported [7]. However, information on placental or breastmilk transfer of these drugs are insufficient.

This report presents the case of a mother who used concomitant psychoactive drugs, including brotizolam, periciazine, sulpiride and zolpidem during the pregnancy and lactation, and alprazolam during lactation, and focuses on the outcomes for her neonate. The discussion addresses the safety of these drugs with reference to their concentrations in cord blood, maternal serum, and breast milk.

## Case presentation

A 38-year-old woman (weight 40 kg) was evaluated for her first pregnancy. At age 28 years, she was diagnosed with dissociative disorder and persistent dysthymia depression, for which treatment with brotizolam (0.25 mg/day), propericiazine (10 mg/day), and zolpidem (5 mg/day) was initiated and continued during pregnancy. Propericiazine was initiated when she was 28 years old, and both brotizolam and zolpidem has been treated for five years.

At gestational week (GW) 28, zolpidem was discontinued. During GW 31, mirtazapine (15 mg/day) was initiated, but was discontinued after 2 weeks due to her symptoms of frustration and fatigue. During GW 33, sulpiride (100 mg/day) was initiated, and her depression and anxiety symptoms were slightly improved.

At GW 38, a healthy normal-birth-weight male neonate was delivered by Caesarean section. The Apgar scores at 1 and 5 min were both 8. No congenital anomalies were observed. Due to persistent post-natal respiratory distress, he was immediately administered oxygen with continuous positive airway pressure. At 4 h postpartum, he was admitted to the neonatal care unit due to bradycardia and decreased oxygen saturation on room air (60–80%) when crying.

Within 24 h postpartum, the neonate’s respiratory condition improved, and oxygen was discontinued. At 72 h postpartum, tachypnea and vomiting developed. The total Neonatal Withdrawal Score was 3, based on the checklist by Isobe et al. [8], a simplified version of the Finnegan checklist that is widely used in Japan. No medication or circulatory support was needed during admission.

Until discharge on postpartum day 7, the infant received both breastmilk and milk formula. Daily milk intake was ranged from 10 to 150 mL for pumped breastmilk and from 30 to 400 mL for milk formula. After discharge, he was bottle-fed formula without breastfeeding at least for 1 month. The mother continued taking brotizolam (0.25 mg/day), propericiazine (10 mg/day), and sulpiride (100 mg/day) postpartum, and alprazolam (0.4 mg/day) was initiated at postpartum day 5. However, at postpartum day 18, sulpiride was discontinued because galactorrhea due to hyperprolactinemia was noted, and sulpiride was replaced to paroxetine (10 mg/day) for treating her depression symptom. For her current anxiety symptoms, lorazepam (0.25 mg) was also initiated at postpartum day 18, as a daily treatment. At the 3-month postpartum health checkup, the infant had no detectable neurologic anomalies.

After ethics committee approval and receiving the mother’s written informed consent, cord blood, maternal serum, and breast milk were collected. Brotizolam, sulpiride, and periciazine (a metabolite of propericiazine) concentrations in maternal serum and breast milk, and alprazolam levels in breast milk were evaluated. The calibration curves for the assay were linear over the range of 0.1–50.0 ng/mL for alprazolam, brotizolam, and periciazine, and 1.0–500.0 ng/mL for sulpiride, and the lower limit of quantitation for each drug was 0.1 ng/mL for alprazolam, brotizolam, and periciazine, and 1.0 ng/mL for sulpiride. The intra-day precision expressed as coefficient of variation ranged from 2.7 to 9.8% for alprazolam, 1.4 to 8.2% for brotizolam, 0.8 to 8.1% for periciazine, and 3.1 to 8.9% for sulpiride. The inter-day precision values ranged from 2.9 to 9.6% for alprazolam, 1.5 to 8.6% for brotizolam, 3.2 to 9.1% for periciazine, and 3.2 to 8.9% for sulpiride. The accuracy values of the assay varied from 96.7 to 103.5% for alprazolam, 95.3 to 104.2% for brotizolam, 95.9 to 105.5% for periciazine, and 95.9 to 104.7% for sulpiride.

Table 1 shows the assay results. In cord blood collected immediately after delivery, brotizolam (0.1 ng/mL) and sulpiride (122.1 ng/mL) concentrations were detected at 19.1 h after each last dose, whereas no periciazine was detected (< 1.0 ng/mL).

**Table 1:** Antipsychotic drug concentrations in serum and breast milk samples

Drugs	Sample types	Postpartum days	Time after dose (hr)	Dose (mg)	Concentration(ng/mL)
Brotizolam	Cord blood	0	19.1	0.25	0.1
	Mother serum	0	33.0	0.25	Below LOD
	Mother serum	5	7.5	0.25	5.0
	Mother serum	17	19.5	0.25	0.3
	Breast milk	9	7.5	0.25	Below LOD
	Breast milk	9	11.5	0.25	Below LOD
Periciazine	Cord blood	0	19.1	10	Below LOD
	Mother serum	0	33.0	10	Below LOD
	Mother serum	5	7.5	10	6.1
	Mother serum	17	19.5	10	Below LOD
	Breast milk	9	7.5	10	0.3
	Breast milk	9	11.5	10	0.1
Sulpiride	Cord blood	0	19.1	100	122.1
	Mother serum	0	33.0	100	63.9
	Mother serum	5	7.5	100	366.0
	Mother serum	17	19.5	100	198.5
	Breast milk	9	7.5	100	445.8
	Breast milk	9	11.5	100	129.2
Alprazolam	Cord blood	0	–	0	Pre-dose
	Mother serum	0	–	0	Pre-dose
	Mother serum	5	7.5	0.4	2.3
	Mother serum	17	19.5	0.4	1.0
	Breast milk	9	7.5	0.4	0.9
	Breast milk	9	11.5	0.4	0.5

*LOD* limit of detection

In breast milk, alprazolam (0.9 ng/mL), sulpiride (445.8 ng/mL), and periciazine (0.3 ng/mL) concentrations were noted at 7.5 h after the last dose on postpartum day 9, whereas brotizolam was not detected. Although each level of the detected drug may not reflect the maximum concentration [9], and these calculations from one or two random milk levels are not justified, the calculated daily infant dose based on average breast milk intake (150 mL/kg/day) was 0.14 μg/kg/day for alprazolam, 66.9 μg/kg/day for sulpiride, and 0.05 μg/kg/day for periciazine. The relative infant doses via breast milk were 1.4, 2.7 and 0.02% of the maternal daily dose, respectively.

## Discussion and conclusion

Brotizolam and sulpiride were detected in the cord blood after delivery. Previous studies reported that brotizolam was not detected in cord blood at 9.2 h after the last dose [10]; in our case, however, a small amount of brotizolam was detected, which parallels the findings for other benzodiazepines [11]. Sulpiride was also detected in the cord blood in our case, and this result agrees with a previous ex vivo study showing that a small amount of sulpiride may cross the human placenta and that several transporters are involved in the bidirectional transport of sulpiride between the placenta and fetal blood [12]. The penetration ratio for brotizolam and sulpiride, were calculated by dividing the concentrations in umbilical cord blood by the maternal serum concentrations at the same time after the last dose (at approximately 19 h), was 33.3% (0.1/0.3) and 61.5% (122.1/198.5), respectively. However, these calculated values lack reliability because the values from the maternal serum samples collected at 17 days postpartum were used. Excretion of alprazolam was reported previously [13–15]. It should be noted that alprazolam concentrations in maternal serum were not considered at steady state, and an accurate transfer ratio of alprazolam into breast milk might not be determined. Sulpiride has been found in breastmilk, and our result aligns with those in previous studies [9, 16, 17] and in a study on breastmilk secretion of amisulpride, a benzamide derivative with sulpiride [18]. Although brotizolam was secreted into breastmilk in previous study [10, 15], it was not detected in our study, possibly due to the different breastmilk sampling time.

Regarding pregnancy outcome, the mother received brotizolam and propericiazine during pregnancy, zolpidem from the first to second trimester, and sulpiride from GW 33 to delivery, with no evidence of congenital anomies for the neonate. The neonatal withdrawal syndrome in our case was not severe, which was in line with the findings of previous observational study of neonates delivered by mothers taking psychotropic or anticonvulsant drugs [19].

Because neonates exposed to periciazine and benzodiazepines during the pregnancy are at increased risk of withdrawal symptoms at birth, in addition to neonatal agitation, hypertonia, hypotonia, tremors, somnolence, and respiratory distress [11, 20], the causal relationship between use of these drugs during pregnancy and neonatal respiratory distress could not be ruled out.

This study has several limitations. First, we could not collect the breastmilk sample at the peak concentration of drugs (approximately 2 h after dose) [9, 15], and an accurate evaluation on maximum drug exposure could not be conducted. Second, the sampling number of maternal serum and breastmilk was small, and drug exposure could not be evaluated by using infant’s serum sample. Third, placental transfers of drugs were evaluated using the postpartum serum sample. Pharmacokinetic profiles of drugs during pregnancy and postpartum may differ; thus, further study is needed.

To the best of our knowledge, this report is the first describing the transfer of sulpiride and periciazine across the placenta, and the first describing the transfer of periciazine across the breastmilk. Although antipsychotic drug use during pregnancy and lactation may have a certain risk for the fetus and breastfed infant, continuing antipsychotic medication may outweigh these risks if the mothers have uncontrolled psychotic symptoms. Further studies are needed to evaluate potentially harmful effects after exposure to benzodiazepines and antipsychotic drugs in utero and during breastfeeding.

## Data Availability

The datasets used and/or analyzed during the current study are available from the corresponding author on reasonable request.

## Abbreviations

GW: Gestational week
